# Outcomes of intravitreal bevacizumab and macular photocoagulation for treatment of diabetic macular edema in a tertiary care eye hospital, Karachi

**DOI:** 10.12669/pjms.335.13222

**Published:** 2017

**Authors:** Adil Salim Jafri, Aziz-ur-Rehman, Abdul Haleem Mirani, Saleh Memon

**Affiliations:** 1 Adil Salim Jafri, Department of Ophthalmology, Al-Ibrahim Eye Hospital, Karachi, Pakistan; 2 Aziz-ur-Rehman, Department of Ophthalmology, Al-Ibrahim Eye Hospital, Karachi, Pakistan; 3 Abdul Haleem Mirani, Department of Ophthalmology, Al-Ibrahim Eye Hospital, Karachi, Pakistan; 4 Saleh Memon, Isra Ophthalmic Research & Development Center, Al-Ibrahim Eye Hospital, Karachi, Pakistan

**Keywords:** Anti-VEGF, Central macular thickness, Diabetic Macular edema

## Abstract

**Objective:**

To study the outcomes of intravitreal injection of Bevacizumab and laser photocoagulation in the treatment of diabetic macular edema (DME).

**Methods:**

Seventy-two eyes of 59 patients with diabetic macular edema were divided into two groups of 41 eyes (Group-A) and 31 eyes (Group-B). Subjects in group-A were treated with three intravitreal injections of Bevacizumab (IVB), and that of group-B with macular photocoagulation. Duration of study was 9 months. Follow up pattern for both groups was1, 2, 3 and 6 months. Best Corrected Visual acuity on log MAR (BCVA) for distance as well as near in each visitwas recorded. Retinal OCT for central macular thickness (CMT) was performed on baseline. SPSS version 20.0 was used to analyze the data.

**Results:**

Mean age of the patients was 53.76 ± 8.82 ranging to 36-71 years. Out of 59 patients, 40 (67.8%) were male and 19 (32.2%) female. It was observed that the difference of results among both groups was not significant. Fig.2 documents visual acuity recorded as Improved; Stable and Worse.

**Conclusion:**

The improvement in BCVA was significant at 6 months in both treatments. The final improvements in BCVA has been almost similar between both the treatment groups although it was noted that IVB group showed early improvement in BCVA at follow-ups of 1 and 3 months. A long term follow-up is required in these cases to see the effect of both these treatment strategies.

## INTRODUCTION

Diabetic retinopathy (DR) is the frequent microvascular complication of diabetes mellitus, affecting 7.7 million people globally^1^,^2^ and is the common cause of blindness among people of working age in the developed world.^3^,^4^ Chances of DR in diabetic adult’s ≥40 years old are 28.5% and risk of vision loss is 4.4% in United States.^5^ Studies originating from Pakistan have reported variable prevalence of retinopathy ranging from 15.7 to 55%.^6^-^8^ Gaddap study reported 27.43% patients with DR and 7.51% with sight threatening diabetic retinopathy (STDR) requiring urgent intervention for vision threatening complications.^9^ Laser photocoagulation became the standard treatment for DR including DME after publication of results from the Early Treatment Diabetic Retinopathy Study in 1990.^10^-^13^

“The Royal College of Ophthalmologists’ Clinical Guidelines for Diabetic Retinopathy” recommends laser alone if patients’ compliance is doubtful.^14^ Introduction of vascular endothelial growth factor inhibitors (anti-VEGF) have changed the scenario of the treatment especially of DME,^15^ there is marked shift towards use of anti-VEGF.^16^ Availability, cost, safety, need for repeated injection and strict monitoring and follow-up compliance of 21.2%^17^ does not make drug therapy a favorable choice for treatment of DME. Purpose of this study was to evaluate the effect of IVB and standard macular photocoagulation (MPC) in management of DME.

## METHODS

This quasi-experimental study was conducted at Al Ibrahim Eye Hospital (AIEH) from April 2015 to December 2015. Approval of this study was obtained by ethical/research committee of Isra Postgraduate Institute of Ophthalmology. Patients with Type-2 Diabetes mellitus of either gender, ≥ 35 years age group, having macular edema (DME) > than 250µ confirmed by retinal Optical Coherent Topography (OCT) was recruited for the study. Fundus Florescence Angiography was done to identify margins of foveal avascular zone, to rule out macular ischemia and determine the boundaries for macular grid laser application. Patients were randomly selected for both the treatment however; choice was given to the patient for opting any of the two treatments. Those who were having cardiac or cerebro-vascular problem were placed in Group-B. Sampling method carried out was “Non-probability, purposive” type.

Informed consent was taken after explaining the pros and cons of laser application and intra-vitreal injection. Seventy-two eyes of 59 patients were included in the study.13 subjects were treated bilaterally and 46 received unilateral treatment MPC was used in 31 eyes and intra-vitreal injection in 41 eyes.

After taking history, “Best corrected visual acuity” (BCVA) was taken at the time of recruitment. Detailed ocular examination was performed, a retina-trained ophthalmologist with slit lamp and dilated fundus was examined with 90D (Volk) lens FFA and retinal OCT was done using 3D OCT – 2000 FA plus by a trained technician. Central Macular Thickness (CMT) measured as mean thickness on the 1-mm circle centered on the fovea. BCVA was taken on each follow up visit and entered into database, at one month, second month, third month and sixth month follow up visits.

Group-A received a total of three-intravitreal injections of bevacizumab (Genentech), 1.25 mg / 0.05 ml at one monthly interval. After ensuring proper sterilization; injection given through the inferior-temporal pars plana, 4 mm from the limbus in phakic and 3.5mm in pseudo-phakic eyes. All the injections were given by the principle investigator. After the injection patients were prescribed topical moxifloxacin 0.5% eye drops four times a day for one week.

Group-B received laser treatment on Pascal double frequency YAG laser. Modified grid laser photocoagulation was performed delivering 2 to 3 rows of 100-micron spots, 100 micron apart in the para-foveal region. Then, 150 to 200 micron spots were applied 200 micron apart to the remaining areas of retinal thickening and capillary non- perfusion. Focal leaks outside or within the zones of diffuse leakage were treated with 100 to 150 micron spots to achieve a mild whitening of the micro-aneurysms. No patient received Pan retinal photocoagulation.

- Follow up pattern for both the Groups A&B was 1 month, 2, 3 and 6 months.
- BCVA was recorded on each visit.

### Main Outcome Measures

The change in best-corrected visual acuity (BCVA) at the end of 6 months considered as “primary outcome”(functional)

### Statistical Analysis

The data was analyzed through the software SPSS version 20.0. The entire continuous variable w presented in Mean ± Standard Deviation. The entire categorical variable was shown in frequency and Percentages. To see the significance between the groups at different visits of baseline to 1^st^, 2^nd,^ 3^rd^& 6^th^ monthly intervals for BCVA. Paired sample t-test was applied. P-value ≤0.05 considered statistically significant.

## RESULTS

This study consisted of 59 diabetes Type-2 patients (72 eyes) Mean age of the patients was 53.76 ± 8.82 with range of 36-71 years. Out of 59 patients, 40 (67.8%) were male and 19 (32.2%) were female. Respondents were divided in two groups. Group-A included 41 eyes who received Intravitreal Injection and Group-B had 31 eyes who received laser application.

**Fig.1:**
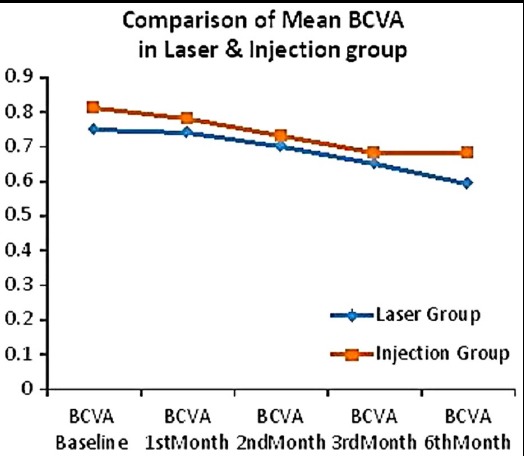
Mean BCVA in both groups at different follow-up. *BCVA=Best Corrected Visual Acuity

**Fig.2:**
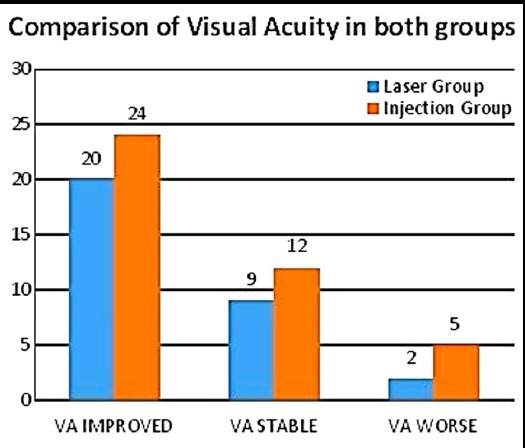
Comparison of Visual Acuity. *VA= Visual Acuity.

This study of eyes with DME showed that therapy with IVB at 3 months appeared to be superior to MPC in improving visual acuity. The improvement in BCVA in both groups was statistically equal and significant at 6 months (Table-I). There was a significant difference found in mean BCVA in both groups while comparing their follow-up with significant P-values 0.001, 0.000, 0.000, 0.000 (Fig.1). Visual acuity which was recorded in terms of improved, stable and worse is shown in Fig.2.

**Table-I:** Mean BCVA comparison between the groups.

*Factor (Laser =31 & Injection = 41)*		*Mean*	*Std. Deviation*	*P-value*
BCVA Re Distance Pre Treatment 1st Visit Re Laser	Laser Group	0.7542	0.30041	0.391
	Injection Group	0.8137	0.28062	
BCVA.R.D. 1^st^ Month Laser	Laser Group	0.7458	0.31049	0.618
	Injection Group	0.7815	0.29029	
BCVA.R.D. 2^nd^ Month Laser	Laser Group	0.7029	0.32842	0.634
	Injection Group	0.7395	0.31662	
BCVA.R.D. 3^rd^ Month Laser	Laser Group	0.6500	0.33926	0.626
	Injection Group	0.6883	0.31962	
BCVA.R.D. 6^th^ Month Laser	Laser Group	0.5987	0.36368	0.259
	Injection Group	0.6890	0.30877	

## DISCUSSION

Present study showed functional improvement in injection group and laser group at the end of 6 months. There were 24 eyes which were improved in injection group and 20 eyes in laser group, 12 eyes were stable and 9 eyes in laser group were stable while BCVA in 5 eyes in injection group were worsen and BCVA in 2 eyes in laser group were deteriorate (Fig.2).

BOLT study found that intravitreal bevacizumab has a greater effect than macular laser treatment in patients with center-involving persistent CSME. At 12 months, there was a significant difference in the mean BCVA (*P* = 0.0006). At 2 years, the mean BCVA was also increased in the bevacizumab group compared to the macular laser therapy group (*P* = 0.005).

In the present study there was a significant difference found in both groups while comparing their follow-up. Subjects were with 24 eyes showed improved vision in injection group and 20 eyes in laser group, 12 eyes were stable in injection group and 9 eyes in laser group, while 5 eyes in injection group were deteriorate and 2 eyes in laser group were deteriorate (Fig.2) From 1^st^, 2^nd^, 3^rd^ & 6^th^ month BCVA improved at follow up with significant P-values 0.001, 0.000, 0.000, 0.000 (Fig.1). Improvement in vision was seen early with IVB at 1^st^ and 2^nd^ month while other group started to show improvement at 3^rd^ month. Both groups have almost statistically equal improvement of BCVA at 6 months.

Masoud Soheilian et al. reported that Intravitreal bevacizumab injection in patients with DME yielded a better visual outcome at 24 weeks compared with macular photocoagulation.^26^ Danial kook MD et al. reported that in cases with chronic diffuse ischemic diabetic macular edema (a long-term decrease of central retinal thickness) can be observed following repeated intravitreal injections of bevacizumab, he also added that “treatment with bevacizumab at an earlier stage of diabetic macular edema without ischemia may be associated with an even better functional outcome”.^27^

In this study, we also found that both IVB and MPC treatments improve VA of DME eyes, at 1^st^ post op. month VA with IVB being significantly superior to MPC. This superiority of VA with IVB appears to wane over longer follow-up periods. At other follow-up points the significant difference was not observed in VA may be limited by effective time of bevacizumab, because its half-life in the eyes is only 9.8 days. The effectiveness of IVB on VA was greater in patients with macular edema in an early follow-up period as indicated in study by Yilmaz et al.^28^ Pharmacokinetic data suggesting a single intravitreal injection of 1.25mg bevacizumab is effective for 6-7 weeks.^29^ Limitations of IVB include regression of visual acuity within a few weeks after treatment, indicating the need for more frequent injections. Studies have reported that MPC had the ability to stabilize VA in long term and it had a significant improving effect on VA.^30^ Considering medical expenses, MPC appear to be more acceptable for the majority of DME patients, especially those in under privileged countries.

Masoud Soheilian et al. reported that Primary outcome measure was change in best-corrected VA (log MAR) at week 24. Secondary outcomes were VA changes at 6, 12, and 36 weeks, as well as CMT changes by optical coherence tomography and potential injection-related complications.^26^ In this study no significant difference was found in both the groups (Table-I).

## CONCLUSION

This study of eyes with DME showed that therapy with IVB at 1 and 3 months appeared to show better improvement in best corrected vision than the laser however both modes of treatment are found to improve BCVA equally at the end of six months. Further studies over a period of 2-3 years are needed to see if the initial benefit is sustained over the time. This was a six month follow up study in order to determine stability in both vision BCVA, a long term follow up study is recommended. Sample size of this study is rather small to see the significance in difference of effect of the treatment.
